# Magnesium Oxychloride Cement-Based Heavyweight Mortars with Coarse Hematite and Barite Aggregates for Gamma Radiation Shielding: Mechanical, Thermal and Microstructural Evaluation

**DOI:** 10.3390/ma19184013

**Published:** 2026-09-21

**Authors:** Bekir Oruncak, Şemsettin Kılınçarslan, Aycan Şengül, Yasemin Şimşek Türker, Nuri Işıldar, İskender Akkurt

**Affiliations:** 1 Department of Physics, Afyonkocatepe University, Afyonkarahisar 03200, Turkey; 2 Faculty of Engineering and Natural Sciences, Suleyman Demirel University, Isparta 32260, Turkey; 3 Medical Imaging Techniques Vocational School of Health Services, Akdeniz University, Antalya 07058, Turkey; 4 Natural and Industrial Building Material Applied Center, Suleyman Demirel University, Isparta 32260, Turkey

**Keywords:** sorel cement, hematite, barite, heavy mortar, thermal, radiation-shielding properties

## Abstract

Magnesium oxychloride cement (MOC) is a non-hydraulic cement that is created through a chemical reaction between light-burned magnesia powder and a magnesium chloride (MgCl_2_) solution. This type of cement has excellent mechanical and physical qualities, such as superior workability, elevated early strength, and reduced density. It is also used in fire-resistant coatings. Compared to Portland cement, MOC has better bonding and compressive strength. This study investigated the feasibility of producing heavyweight radiation-shielding mortars using an MOC binder with coarse hematite and barite aggregates of up to 8 mm, which are significantly larger than the fine aggregate sizes traditionally used in MOC systems. The mechanical, thermal, microstructural and gamma-ray performances of the mortars were experimentally evaluated. In this study, two different groups of mortar specimens were prepared using a magnesium oxychloride cement (MOC) binder with natural crushed stone, hematite, and barite aggregates. The thermal conductivity, compressive strength, bending strength and radiation-shielding properties of the produced mortar samples were examined. To overcome the inherent water sensitivity of MOC, a phosphate modification was incorporated into the binder system. A flexural strength, compressive strength, thermal conductivity, density, and scanning electron microscopy (SEM) analysis, and gamma radiation attenuation tests were performed using ^137^Cs and ^60^Co sources at 662, 1173 and 1332 keV. The strength values of MOC samples produced with normal aggregate were higher than those with hematite and barite. The thermal conductivity values of BMOC samples and HMOC samples gave similar results. The thermal conductivity values of mortars produced with normal aggregate were very high. The radiation retention values of barite aggregated samples were higher than those of other samples. The lowest radiation-shielding value was obtained from the normal aggregated samples. The findings demonstrate that phosphate-modified MOC can successfully incorporate coarse heavyweight aggregates of up to 8 mm while maintaining satisfactory mechanical performance and providing enhanced gamma-ray shielding, offering an environmentally promising alternative to conventional Portland cement-based shielding materials.

## 1. Introduction

In modern societies, the use of radiation in various forms and for increasing purposes puts all living things at biological risk. Today, the application of radiation in fundamental science, medicine, agriculture, industry, and military endeavors has attained significant and extensive proportions [1]. Currently, there are over three thousand nuclear facilities globally that serve the needs of medicine, scientific research, energy, agriculture, and industry. Additionally, medical centers use radiation beams for both diagnosis and treatment. Radiation is energy disseminated in discrete units known as waves, particles, or photons. The three fundamental components of radiation protection are the principles of time, distance, and shielding [2]. Including a shielding material between the radiation source and the individual decreases the exposure dose. The effectiveness of shielding against X-rays and gamma rays is dependent on the density of the material used. Heavy aggregate mortar is considered the most appropriate material for achieving this type of shielding [3]. Materials including lead, iron, graphite, water, polyethylene, and mortar have been identified as materials with the potential to serve as effective shields against the harmful effects of nuclear radiation. One of the most effective widely utilized construction materials for shielding against gamma and radioactive radiation is mortar [4,5,6]. The tensile, compressive, and bending strengths of mortar are essential mechanical and physical properties critical for this type of protection [7,8]. Protecting the environment from the damaging effects of radioactive radiation is a paramount concern in the application of nuclear technology [9,10]. Materials scientists and physicists have investigated radiation in order to advance nuclear technology and create new methods that can be widely applied in a variety of sectors. Medical institutions and nuclear plants are always thinking about how to provide and construct mortar that is gamma-ray resistant. The effects of mortar additives and aggregates for gamma-ray protection have been extensively studied [11,12,13,14,15,16]. Ahmed and Hakim [17] looked at using high-mechanical-property local aggregates as gamma-ray shields. Tests were conducted on mortar mixes including varying amounts of components (20%, 40%, 60%, 80%, and 100%). To test the compressive and tensile strengths, two mortar mixes were employed, with 50% and 60% of the heavy fine aggregate used in place of the coarse aggregate. The findings demonstrated that the compressive and tensile strengths decreased when the ratio increased by over 60%.

Richard and Cheyrezy [18] defined and recommended the appropriate mechanical qualities and durability of ultra-high-performance mortar (UHPC) for nuclear and industrial waste. Scientists are working to create a UHPC that can withstand nuclear radiation while preserving its exceptional mechanical and microstructural characteristics [19,20,21]. It has been demonstrated that adding barite instead of quartz aggregate to UHPC mortar may greatly raise the gamma-ray attenuation coefficients [22,23]. Additionally, it was discovered that the flexural strength is negatively impacted when quartz is substituted with barite aggregate. The ideal UHPC combination for concurrent protection against neutrons and gamma rays is one with a compressive strength of 40% barite aggregate (of aggregate volume). Nuclear sites employ mortar that contains hematite and barite, which are excellent radiation-shielding materials [24]. An investigation of the impacts of hematite on UHPC properties was carried out by Lv et al. [25]. The experimental results demonstrated that the incorporation of hematite into the material resulted in a reduction in its compressive strength but, on the other hand, the material demonstrated enhanced strength, with a substantial increase in its flexural and impact strengths. Additionally, its performance at elevated temperatures was augmented. A comparison of the linear attenuation coefficient of UHPC with a 40% hematite replacement ratio revealed an increase of 43%, while the half-value layer’s thickness demonstrated a decline of 30%. Khan et al. [26] looked at using hematite powder in place of sand in UHPC. The minimum dry density taken into consideration was 2600 kg/m^3^ to 2900 kg/m^3^, and this was the density of all of their UHPC blends. These combinations weighed the same as mortar. They said that there was minimal variation in the mechanical characteristics of the UHPC mixes despite the fact that they had varying dry specific gravities. Hematite and barite aggregates were utilized by Azreen et al. [27] in ultra-high-performance (UHPC) systems. According to their findings, the mixture’s toughness increased, making it the perfect radiation absorber for nuclear installations. After 28 days, all of their UHPC samples had reached a high compression strength of greater than 130 MPa. In spite of this, the gamma-ray shielding properties of UHPC with barite aggregates were superior. The basis for this assertion was the chemical interaction between the light-burned magnesia (LBM) powder and MgCl_2_ solution, as MOC is a non-hydraulic cement [28]. The excellent mechanical and physical characteristics of this cement include a low density, strong early strength, and outstanding workability [29]. Additionally, it may be used on coatings that resist fire [30]. MOC has far superior bonding and compressive strength than regular Portland cement (OPC). Additionally, MOC has a lower pH than OPC, which is often higher than twelve [31]. According to Wang et al. [32], the calcination temperature of magnesite used in the cement production process to make LBM is around 800 °C, but the temperature required to manufacture OPC is greater than 1300 °C. MOC, a material regarded as eco-friendly and energy saving, has emerged as a potential alternative to OPC.

This study investigated two separate sets of mortar samples. Initially, mortars utilizing a magnesium oxychloride binder were fabricated with conventional aggregate. Then, MOC-based mortar specimens containing hematite and barite aggregates were prepared. The thermal conductivity, compressive strength, bending strength and radiation protection properties of the produced mortar samples were examined. Due to its general structure, the strength of MOC decreases after exposure to a humid environment and immersion in water. The separation of hydration phases results in the leaching of MgCl_2_, which creates a corrosive effect on the steel reinforcement in mortar. The inadequate water resistance of MOC limits its use in civil engineering. In this study, phosphate was added to MOC to enhance its water resistance and address expansion-related issues. In addition, another feature of the study is that MOC mortar can be produced with aggregates larger than 8 mm. In the second part of the paper, the preparation of materials and samples is mentioned. In the third section, the experimental setups and performance of the experiments are discussed. In the next section, the findings are evaluated, and the obtained data are reconciled with those in the literature. In the last section, the results obtained in the study are emphasized.

Although heavyweight aggregates such as barite and hematite have been extensively investigated in Portland cement-based shielding concretes, their incorporation into magnesium oxychloride cement (MOC) systems remains very limited. Furthermore, previous MOC studies have predominantly focused on fine aggregate systems, while the behavior of MOC mortars containing coarse heavyweight aggregates has not been sufficiently investigated. In addition, the combined evaluation of mechanical performance, thermal conductivity, gamma-ray attenuation, and microstructural characteristics within a phosphate-modified MOC system has rarely been reported. Therefore, the present study aims to fill this gap by investigating the feasibility of producing MOC-based heavyweight mortars incorporating coarse hematite and barite aggregates (up to 8 mm) for multifunctional radiation-shielding applications.

## 2. Materials and Methods

### 2.1. Materials

**Figure 1:**
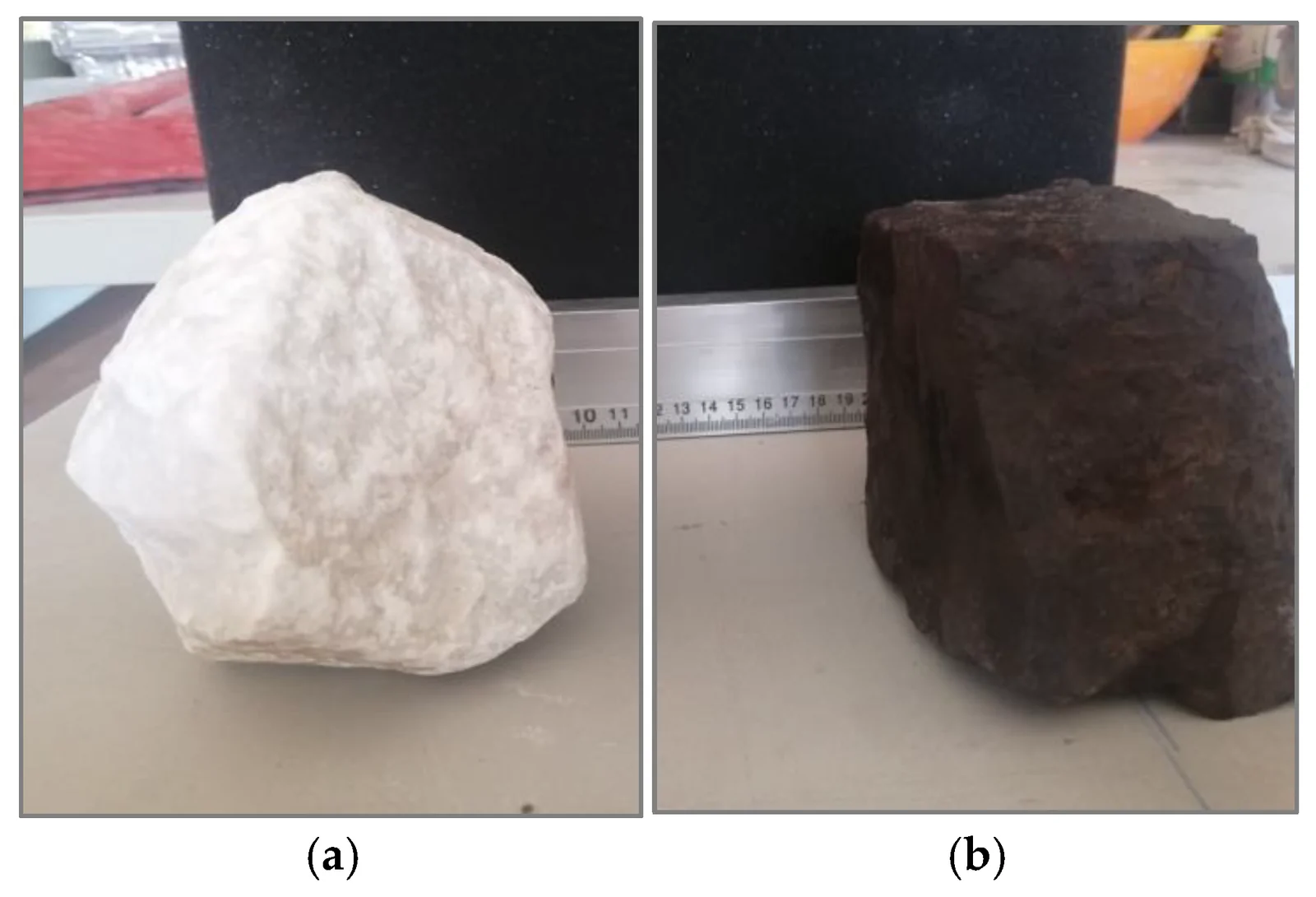
Images of the aggregates: (**a**) barite, (**b**) hematite.

In the context of UHPC research, the selection of materials in the mix design is a critical factor that must be considered. The components utilized in the present study were derived from the findings of preceding research endeavors [33,34,35,36]. Light-burned magnesium oxide (MgO) (TEKKİM Chemical Co., Bursa, Türkiye; catalog no. TK.200920; purity ≥ 88 wt.%) was used as the reactive magnesia source. The magnesium chloride (MgCl_2_) solution was prepared by dissolving MgCl_2_ powder in distilled water before mixing. A polycarboxylate ether-based superplasticizer (PF65) was used to improve workability. In addition, a phosphate-based additive was incorporated into the MgCl_2_ solution to enhance the water resistance of the MOC binder. Hematite, barite and natural crushed stone were used as the aggregates. The hematite and barite aggregates were obtained from different regions of Isparta Sarkikaraagac. In order to standardize the granulometry of the aggregates used, a sieve analysis was performed, and castings were made in accordance with the determined granulometry. Hematite is a high-density, iron oxide mineral that crystallizes in a rhombic lattice system, similar to ilmenite and corundum. The ore’s significant density enables its application as a weighing agent in drilling fluids. The mica’s crystal structure makes it an ideal medium for drilling fluid. Due to its high specific gravity, hematite can be used as an effective barrier against gamma rays in mortar. After crushing, one of the most important step to obtain high strength mortar is a sieve analysis. The size of the aggregates must have a distribution in accordance with the standards, which means that the overall grain distribution must be known, or the aggregate grain distribution ratios must be adjusted correctly to ensure a suitable composition and prevent grain segregation. Images of the barite and hematite aggregates used in the study before grinding are given in Figure 1.

**Figure 2:**
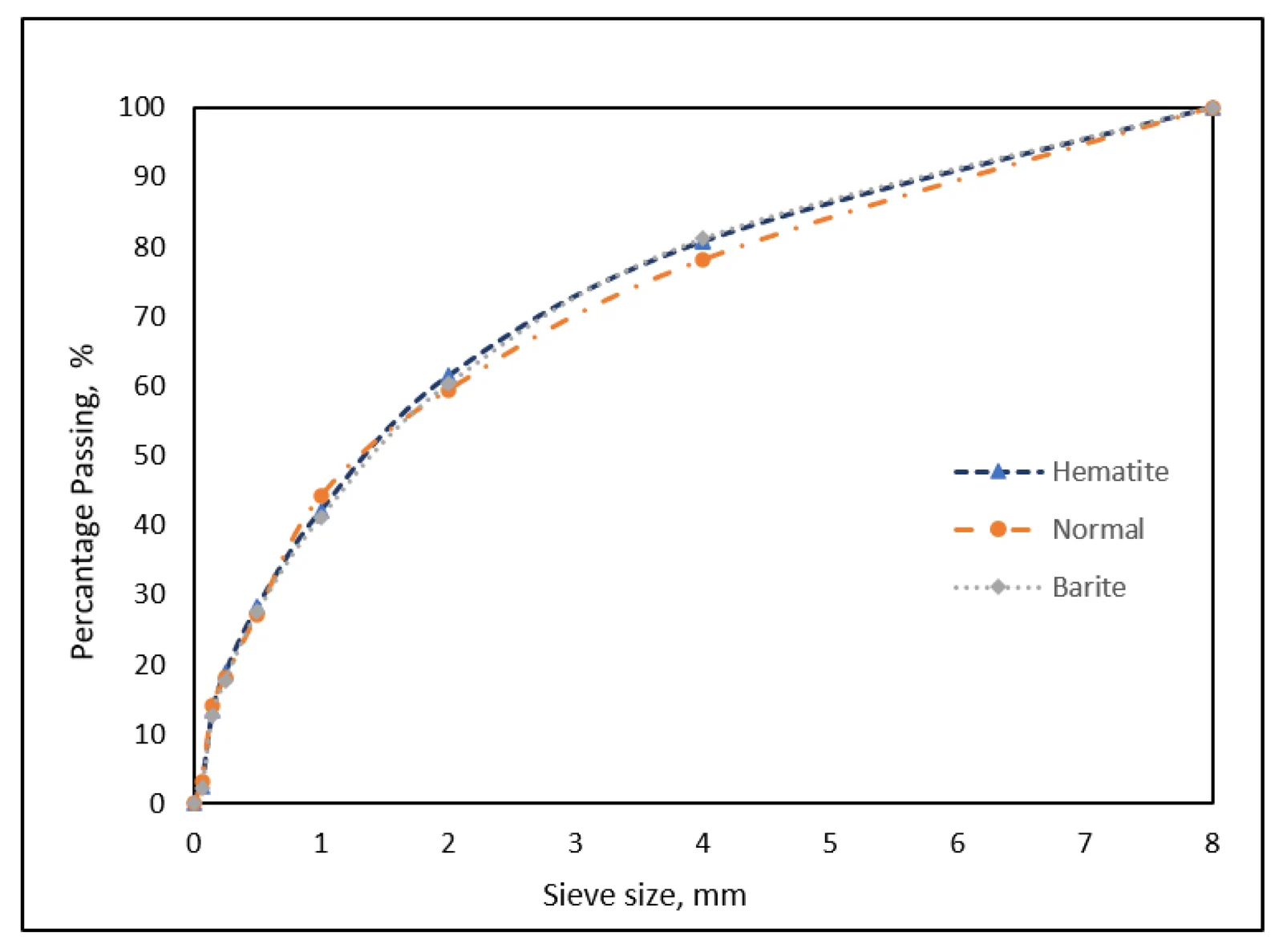
Granulometry curves of aggregates.

This was done to ensure the most suitable formulation, to obtain the appropriate consistency by using the least amount of water, to prevent segregation in the fresh mortar, to ensure good sealing and adhesion, and to reduce the amount of sweating. The aggregates were crushed in a laboratory-type jaw crusher and aggregates in the range of 0–8 mm were obtained. For the grain size distribution, the samples were crushed with the help of a jaw crusher and grain size distribution graphs were obtained. In the sieve analysis conducted in accordance with the TS EN 1015-1 standard [37], the aggregate pieces were weighed with a precision scale, then sieved, and after being separated into sizes, they were weighed again, and the values were recorded. The granulometry curves obtained are given in Figure 2.

### 2.2. Preparation of Specimens

In this study, two different groups of mortar samples were examined. Firstly, mortar with magnesium oxychloride binder were produced using normal aggregate (A). Then, mortars with MOC binder were produced using hematite (H) and barite (B) aggregate. The thermal conductivity, compressive strength, bending strength and radiation protection properties of the produced mortar samples were examined. Due to its general structure, the strength of MOC decreases after exposure to a humid environment and immersion in water. The segregation of hydration phases results in the leaching of MgCl_2_, which induces a corrosive impact on the steel reinforcement within mortar. The inadequate water resistance of MOC restricts its application in civil engineering. In this study, phosphate was added to the MOC to increase its water resistance and solve the expansion-related problems. Another objective of this study was to evaluate the feasibility of producing MOC mortars incorporating heavyweight aggregates with a maximum particle size of 8 mm. The results were compared with heavy mortar with Portland cement binder having the same unit volume mass. The mixture design of mortar samples is given in Table 1.

Mixing Procedure: Prior to mixing, MgO and the selected aggregate were dry-mixed to ensure a homogeneous distribution of the solid constituents. In parallel, MgCl_2_ powder was completely dissolved in distilled water to prepare the magnesium chloride solution. The phosphate-based additive was subsequently dissolved in this solution, followed by the addition of the PF65 polycarboxylate-based superplasticizer. The modified MgCl_2_ solution was then gradually added to the dry mixture and mechanically mixed for approximately 5 min at a constant speed. The fresh mortar was subsequently poured into the molds under vibration to minimize entrapped air and ensure adequate compaction. After casting, all specimens were cured under laboratory air conditions at room temperature for 28 days prior to testing.

**Figure 3:**
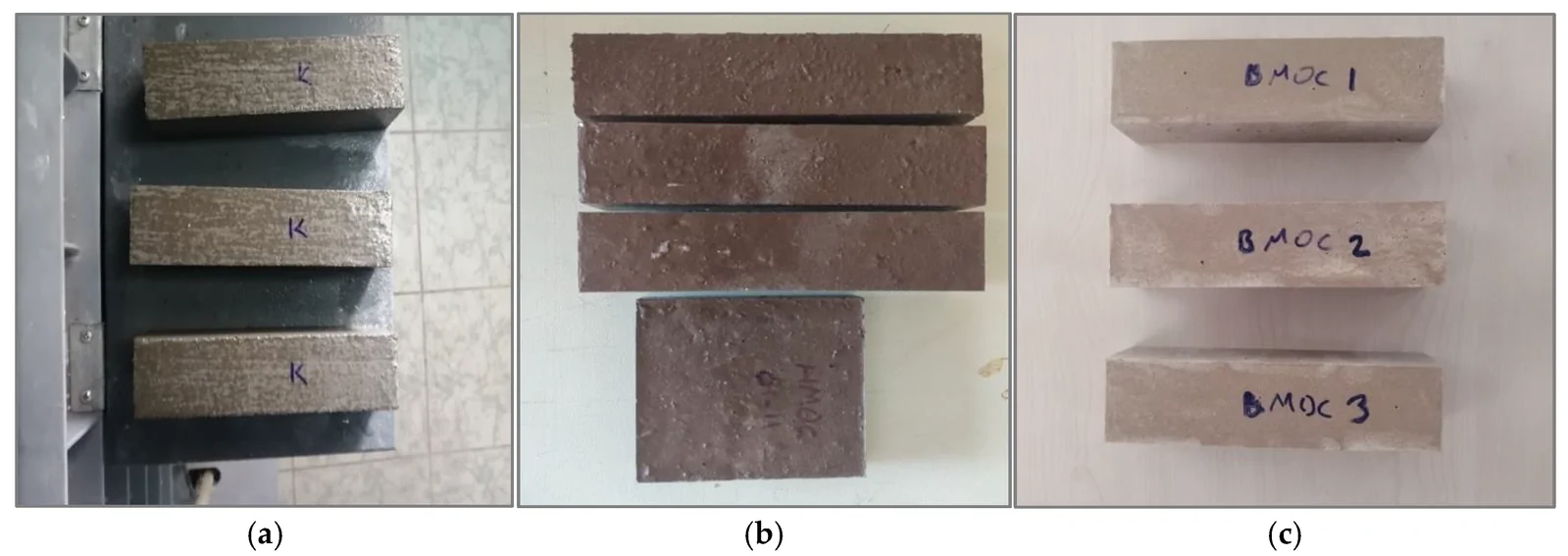
Mortar samples: (**a**) normal aggregate, (**b**) hematite aggregate, (**c**) barite aggregate.

Samples were prepared for flexural strength, compressive strength, thermal conductivity, and radiation attenuation tests. Flexural strength tests were performed on three prism specimens for each mixture in accordance with TS EN 196-1 [38]. After the flexural test, each fractured prism yielded two halves, which were subsequently used for the compressive strength testing, resulting in six compressive strength measurements for each mixture. Representative fragments obtained after the mechanical tests were used for the SEM observations. The amounts of MgO, MgCl_2_, aggregates, water, and chemical admixtures were measured using precision balances before mixing. Mixture calculations were performed considering a maximum aggregate size (d_max_) of 8 mm. The production process of the mortar specimens is illustrated in Figure 3.

### 2.3. Experimental Methodology

The density of the materials utilized in this study was ascertained using a helium pycnometer to formulate a mortar design plan and to elucidate the qualities of the aggregates employed. Flexural and compressive strength determination tests were carried out on the mixtures prepared with the data obtained here, in accordance with the TS EN 196-1 standard. Samples of 40 × 40 × 160 mm were prepared for the determination of bending strength. In this testing method, the bending strength of the samples was measured according to the 3-point loading method. In the compressive strength test, two pieces from the bending test were used. The prism samples were loaded automatically with a loading speed of 2400 n/s.

**Figure 4:**
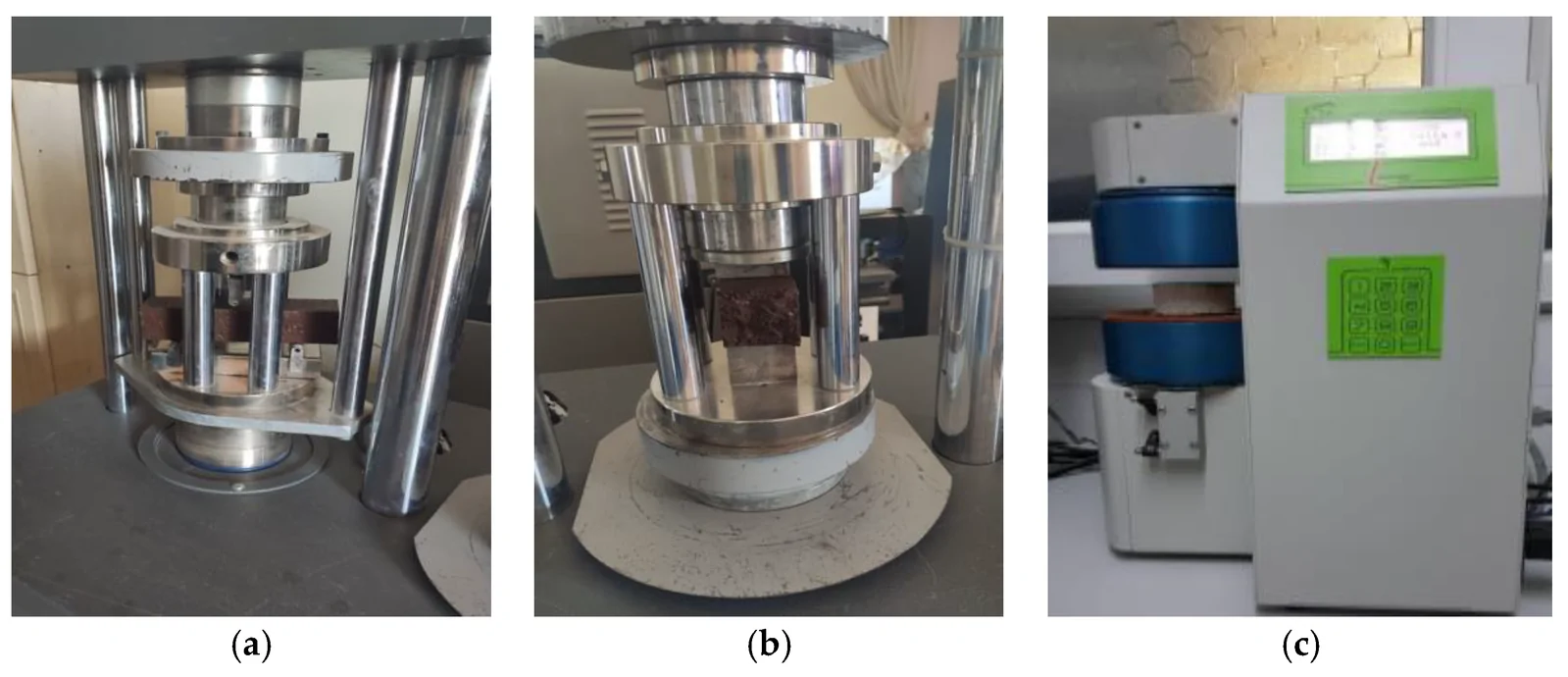
(**a**) Flexural strength, (**b**) compressive strength and (**c**) thermal conductivity tests.

For the thermal conductivity coefficient determination experiment, a Lasercomp Fox 50 thermal conductivity device, which works with the heat flux measurement method, was used at SDU DEYMAM. This device can make measurements in accordance with the TS EN 12667 (2003) standard [39]. It is designed for thermal conductivity coefficient measurements of materials with a thermal conductivity coefficient of less than 10 W/mK. Using this device, the thermal conductivity coefficient (λ) of samples up to 63 mm in diameter and 1–30 mm in thickness can be measured. In the experimental study, one cylindrical sample of each mixture was produced with a diameter of 63 mm and a thickness of 25 mm. Care was taken to ensure that the sample surfaces were flat and smooth. The thermal conductivity tests were carried out based on the TS EN 12667 (2003) standard. The flexural strength, compressive strength and thermal conductivity tests primarily performed on the samples are given in Figure 4, respectively.

The radiation-shielding experiments were carried out in the Gamma Spectroscopy Laboratory. A gamma spectroscopy system is a system that separates the emitted gamma rays based on their energy as a result of their interaction with the NaI (TI) detector. The radiation absorption coefficients were measured using a radiation-shielding experimental setup with a NaI (TI) scintillation detector. A scintillation detector consists of two main parts: the scintillator, which is a scintillating material, and the photomultiplier tube, which contains the photocathode, electrode, electron multipliers, and anode. First, the gamma ray enters the scintillator second undergoes ionization, and excitation occurs because of its interaction with some solid, liquid or gaseous substances called scintillation phosphors [40,41]. When the energy given to the electron excited by the gamma rays is not enough to remove it from its place in the environment, the excited electron returns to its previous state and emits light. The emitted light is collected by photomultiplier tubes and converted into a voltage pulse. The amplitude of this pulse correlates with the energy of the radiation. In these detectors, counting and energy separation can also be done. The signals created by the gamma radiation in the NaI (TI) detector are amplified with the help of an amplifier and shaped to provide appropriate energy separation. The signal coming out of the amplifier is received by the Multi-Channel Analyzer (MCC) containing 16,384 channels, each of which corresponds to an energy, and is converted into digital form here. The information converted into digital form by the analyzer is subsequently observed on the screen as a spectrum. The system is fed by the high-voltage unit.

The radiation absorption properties of mortars were investigated by obtaining the total linear absorption coefficients (μ, cm^−1^) of the mortars. If a gamma radiation beam of intensity I is attenuated by ΔI in an absorber of thickness x,

ΔI = − μ I Δx

If this equation is integrated,

I = I_0_e ^−μx^

is obtained. This equation is also known as the Beer–Lambert equation.

In this equation, I_0_ is the radiation intensity before interaction with the substance, I is the radiation intensity after interaction with the substance, x is the thickness of the absorbing material, and μ represents the linear absorption coefficient.

The linear attenuation coefficient (μ) was determined directly from the experimentally measured transmission data using the Beer–Lambert equation rather than being calculated from the product of the density and the mass attenuation coefficient. The incident intensity (I_0_) was obtained from measurements without the specimens, whereas the transmitted intensity (I) was measured after placing each mortar sample between the radioactive source and the NaI (Tl) detector under identical geometric conditions. Three independent measurements were performed for each specimen, and the average values were used to calculate μ. Consequently, the attenuation coefficients presented in this study represent experimentally measured parameters that inherently account for both the material composition and density.

**Figure 5:**
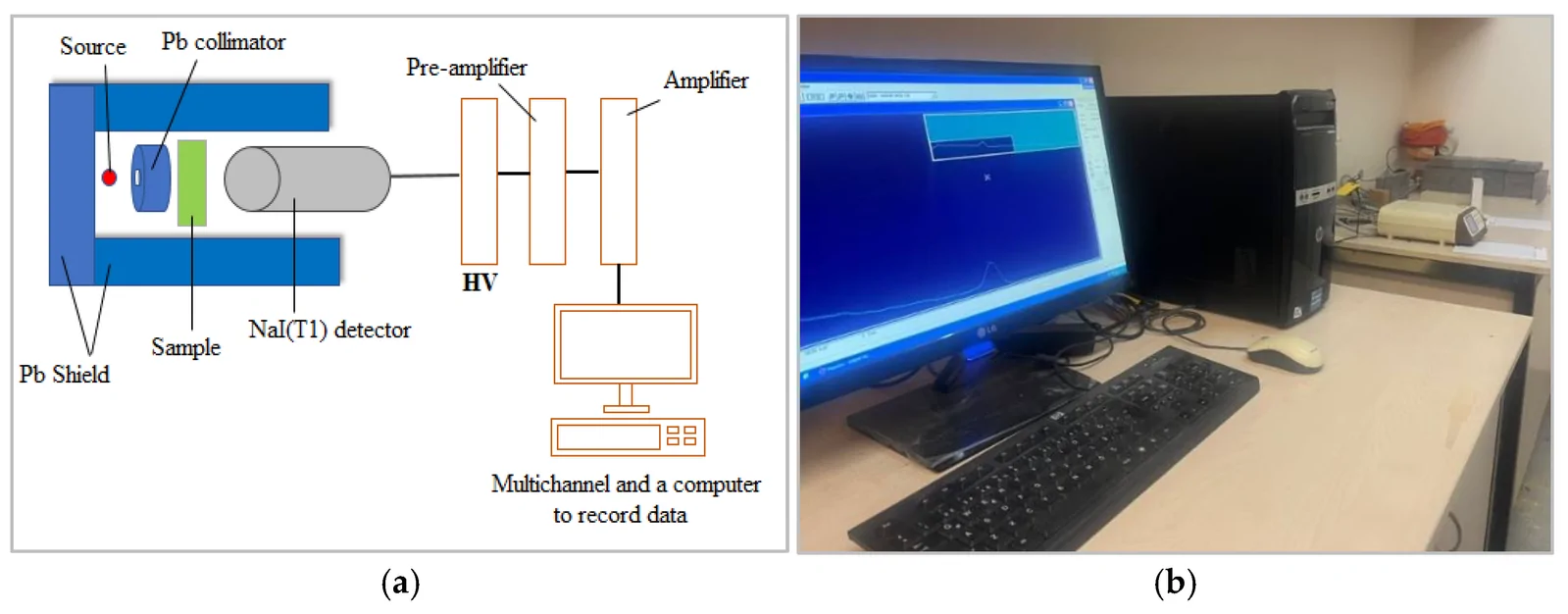
(**a**) Schematic and (**b**) real image of detector and electronic devices that make up the gamma spectroscopy system.

The reference measurement was taken once and used for all mortar specimens. Samples with dimensions of 100 × 100 × 20 mm were prepared, corresponding to a specimen thickness of 20 mm. The samples were then placed between the radiation source and the detector. The radiation absorption coefficients of the mortars were measured at photon energies of 662, 1173, and 1332 keV using ^137^Cs and ^60^Co radioactive sources. During the experiments, each sample was positioned at the same distance from both the radiation source and the detector, as shown in Figure 5.

**Figure 6:**
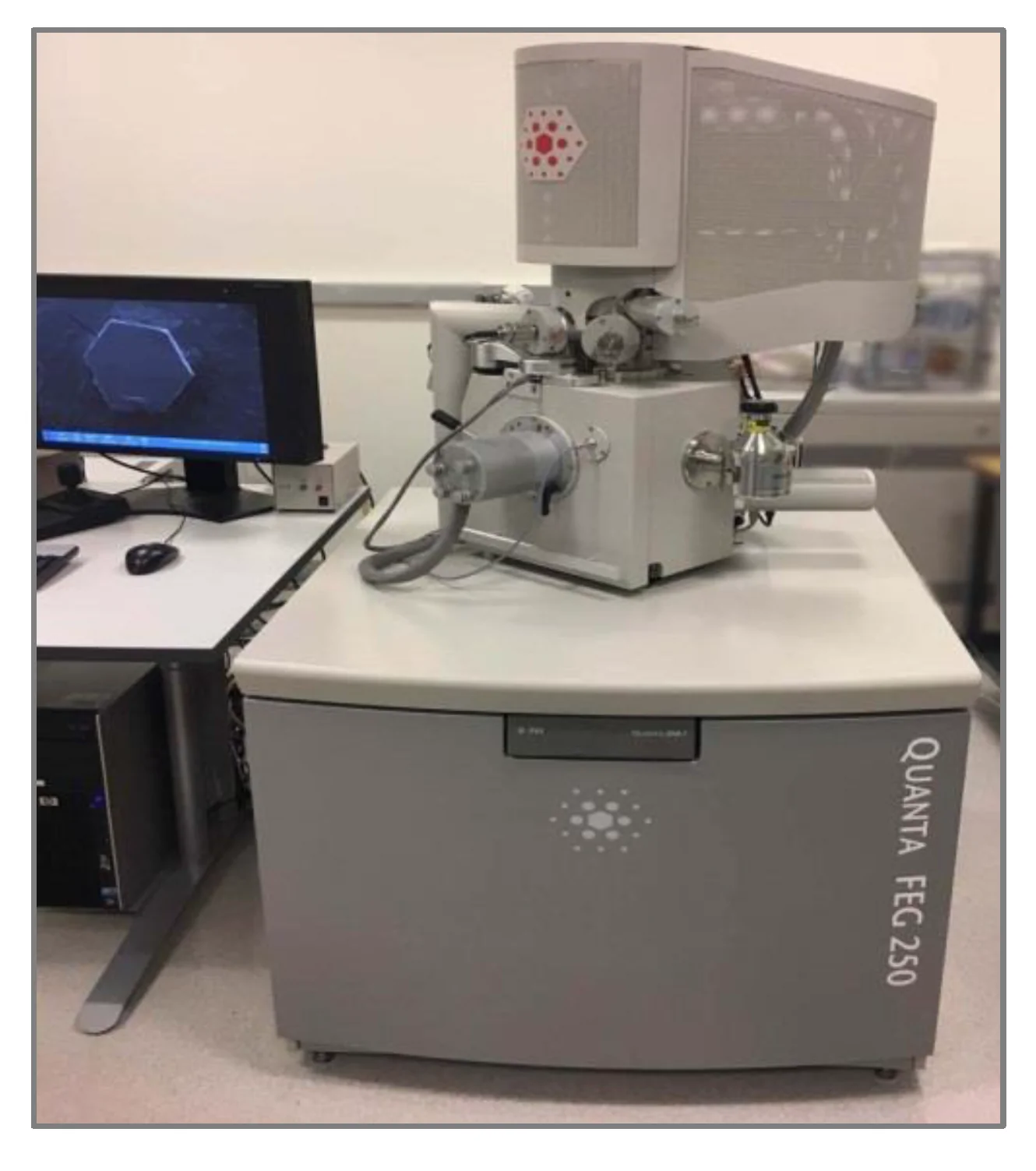
Examining and obtaining images in a scanning electron microscope.

In addition, an SEM analysis of the HMOC and NMOC samples was carried out at the Innovative Technologies Research Center of Suleyman Demirel University (SDU YETEM). The SEM observations were performed using a QUANTA FEG 250 scanning electron microscope (FEI Company, Hillsboro, OR, USA). Most of the micrographs were acquired at an accelerating voltage of 20 kV without a conductive coating (Figure 6). An SEM, one of the basic electron microscopes, takes images with a much better resolution than an optical microscope. The radiation released from the interaction of the electron beam with the materials in the sample creates the SEM images. In this study, the pore distributions, sizes, cell wall thicknesses and whether the voids were connected to each other were examined for the samples.

## 3. Results and Discussion

This study analyzed two distinct sets of mortar samples. Firstly, mortars with a magnesium oxychloride binder were produced using normal aggregate (DA). Then, mortars with an MOC binder were produced using hematite (H) and barite (B) aggregate. The thermal conductivity, compressive strength, bending strength and radiation protection properties of the produced mortar samples were examined. The grain densities and water absorption rates by mass, obtained as a result of the pycnometer tests of the normal aggregate, hematite and barite used in the study, are given in Table 2.

**Figure 7:**
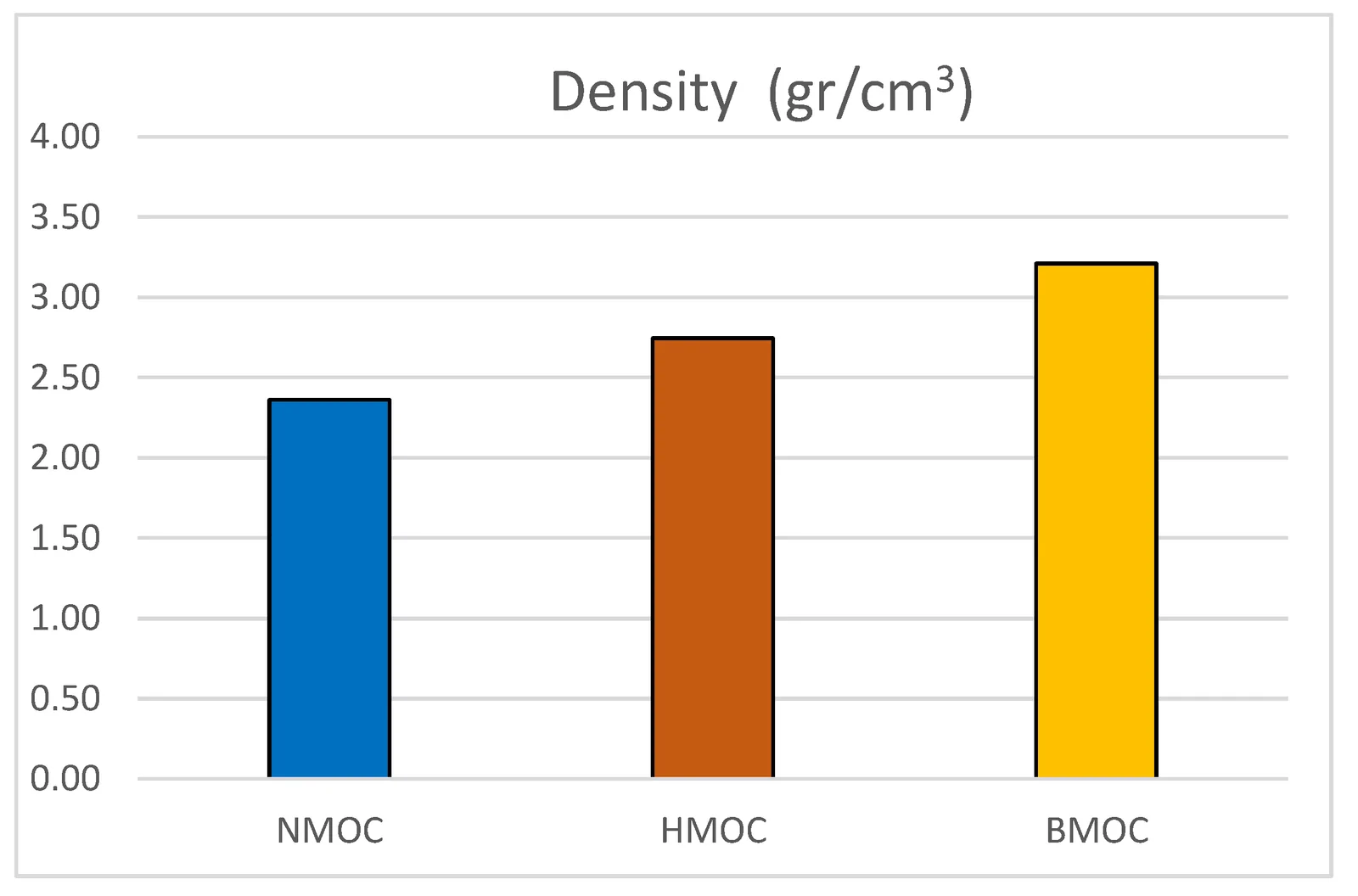
Unit volume weight values of mortar samples.

The graph showing the unit volume weight values of mortar samples is given in Figure 7.

The highest unit volume weight value was obtained for the barite aggregate mortars. The unit volume weight value of the BMOC coded sample was 15% lower than the HMOC coded sample and 27% lower than the NMOC coded sample. The unit volume weight value of the HMOC coded sample was 14% lower than the NMOC coded sample.

**Figure 8:**
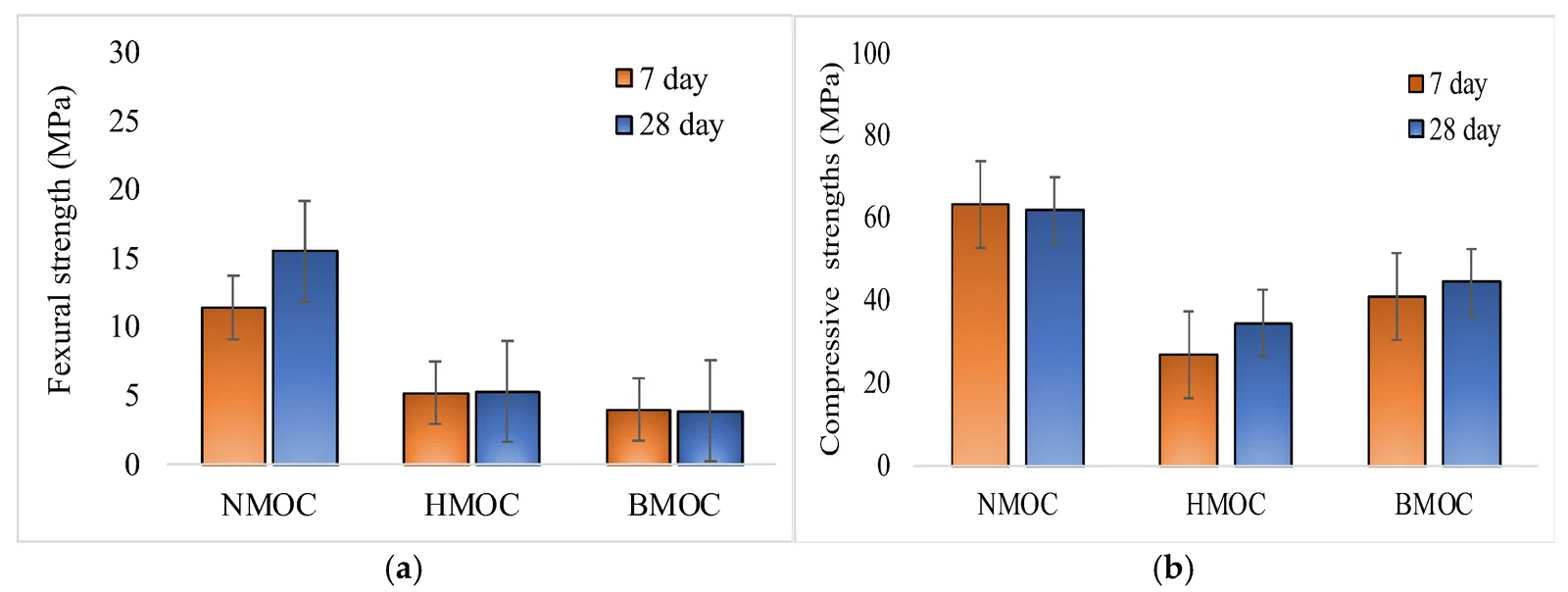
The 7- and 28-day (**a**) bending and (**b**) compressive strengths of mortar samples.

The 7- and 28-day bending and compressive strength values of the mortar samples, whose unit volume weight values were determined, are given in Figure 8.

**Figure 9:**
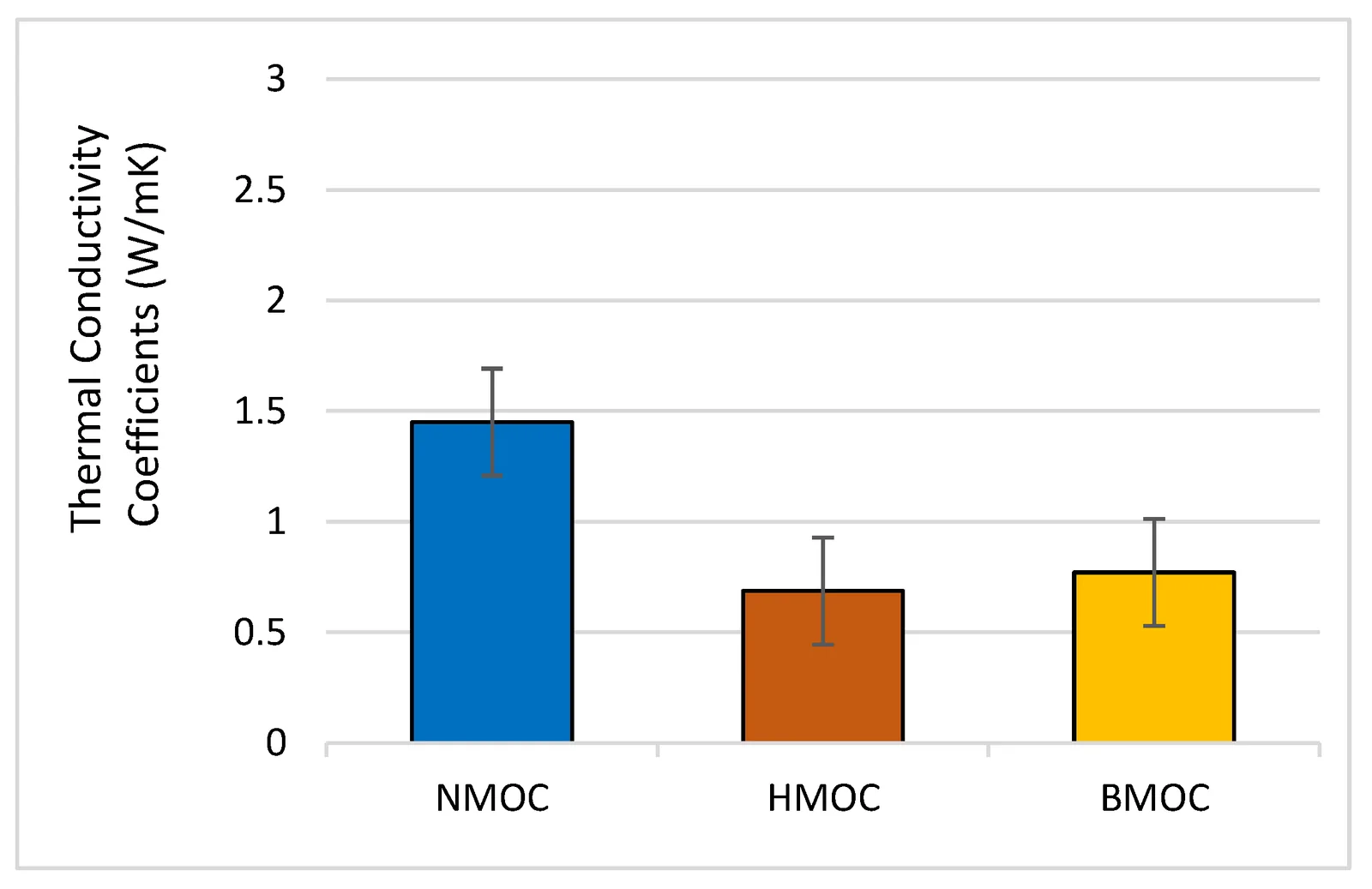
Thermal conductivity values of the produced samples.

The BMOC coded sample had the lowest 28-day bending strength value (4 MPa). The highest bending strength value (11.4 MPa) belonged to the NMOC coded sample. The highest compressive strength value (61.84 MPa) was obtained for the NMOC coded sample. The lowest compressive strength value was obtained for the sample coded HMOC. It was determined that there was a slight decrease in strength values with the use of heavy aggregates. Figure 9 presents the thermal conductivity values of samples created with the three different mixture ratios.

**Figure 10:**
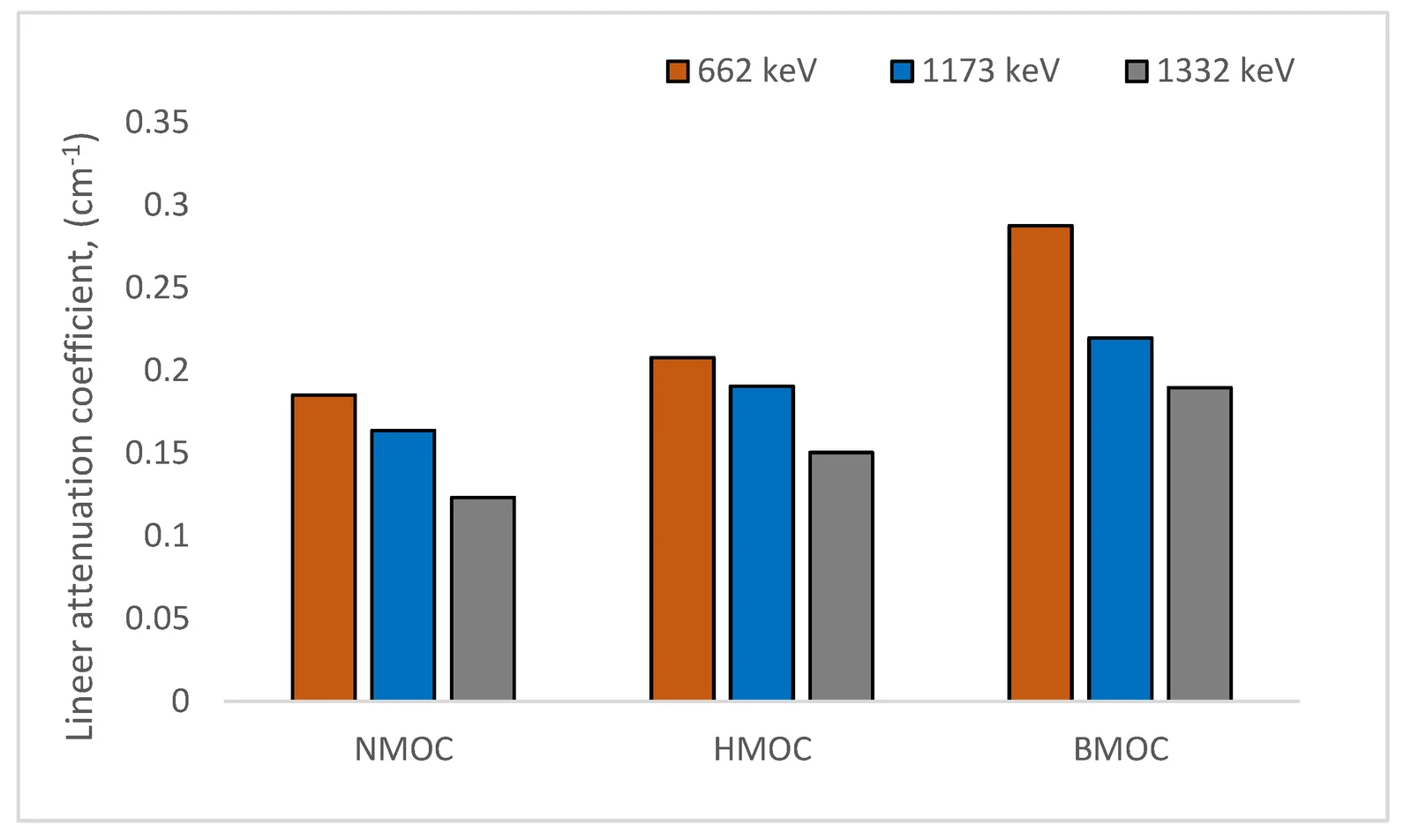
Linear and mass attenuation coefficient values of the produced samples.

The thermal conductivity value of the NMOC coded sample was 53% higher than the HMOC coded sample. The thermal conductivity value of the NMOC coded sample was 47% higher than the BMOC coded sample. It was determined that the thermal insulation of HMOC and BMOC samples increased significantly. In addition to the thermal conductivity, the compatibility of the coefficients of thermal expansion (CTE) between the aggregate and the cementitious matrix is another important factor affecting the thermal durability of cement-based composites. Differences in the CTE may generate localized stresses at the aggregate–matrix interface during heating and cooling cycles, leading to the initiation and propagation of microcracks. Hematite exhibits a linear CTE of approximately 9.2 × 10^−6^ °C^−1^ within 20–200 °C, while barite generally shows lower values ranging from 4.2 to 8.5 × 10^−6^ °C^−1^, depending on the temperature and crystallographic orientation. MOC matrixes have been reported to possess a relatively low thermal expansion compared with conventional Portland cement systems. Consequently, a CTE mismatch between the MOC matrix and heavyweight aggregates may contribute to interfacial thermal stresses under rapid temperature variations. Although no thermal shock or elevated-temperature experiments were conducted in the present study, the observed dense aggregate–matrix contact in the SEM images suggests that the interface quality may partially mitigate the stress concentration under moderate thermal loading. Nevertheless, dedicated thermal cycling experiments are required to quantify the influence of aggregate type on the thermal shock resistance and long-term durability of MOC heavyweight mortars. These findings should therefore be regarded as a mechanistic interpretation rather than direct experimental evidence [42,43,44,45,46,47,48,49,50,51,52,53]. The data obtained is given in Figure 10.

The radiation-shielding value of barite aggregate samples was 54% higher than the normal aggregate samples. The radiation retention value of hematite aggregate samples was 22% higher than the normal aggregate samples. Akkurt et al. [12] examined the radiation-shielding properties of mortar with normal crushed stone aggregate. In this study, the linear attenuation coefficient of mortar produced with normal aggregate was determined as 0.10 cm^−1^ at 1332 keV energy. In this study, the linear attenuation coefficient of normal aggregate mortar produced with magnesium oxychloride cement was determined as 0.13 cm^−1^.

**Figure 11:**
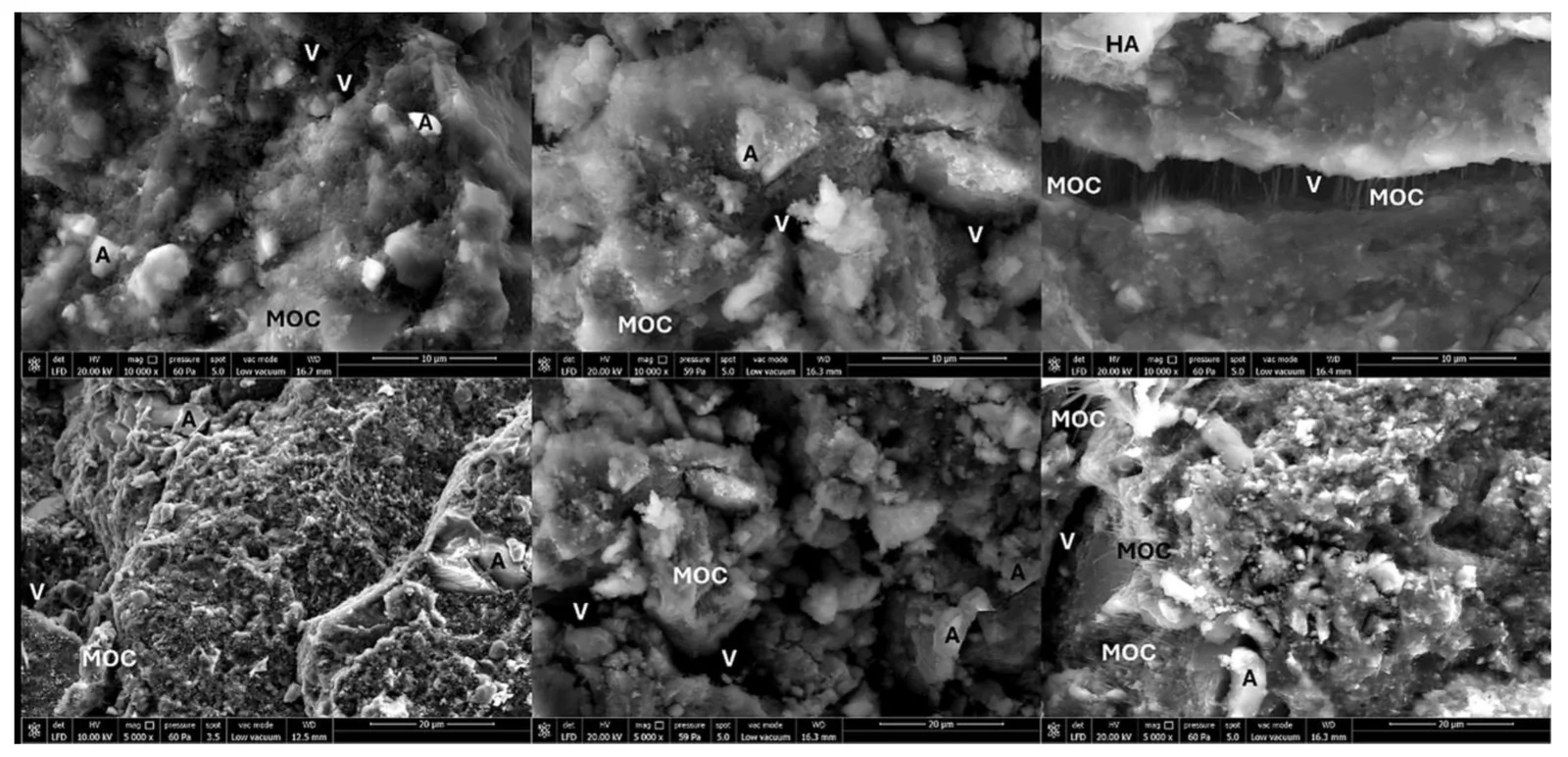
SEM images of BMOC (**left**), NMOC (**middle**) and HMOC (**right**) samples (×5000 and ×10,000) (H: hematite; MOC: magnesium chloride; A: aggregate; V: void).

For the samples obtained within the scope of the study, the void structure, distribution of aggregates and phase formations were examined on the SEM images taken at SDU YETEM. The SEM images presented in Figure 11 clearly demonstrate that the incorporation of coarse heavyweight aggregates fundamentally alters the internal pore architecture and aggregate–matrix interaction within the MOC mortars.

Although there are open pores, the cells are generally in the form of closed pores. Additionally, there are 5Mg(OH)_2_ in all the samples and MgCl_2_. The formation of 8H_2_O (Phase 5) crystals is clearly observed. The SEM imaging of HMOC and NMOC samples shows the formation of magnesium oxychloride. Additionally, hematite particles are also observed in the HMOC samples, which are evident by the glow on the SEM images. These observations indicate that hematite aggregate particles repel each other, creating minimal voids. Hematite aggregates exhibit significant porosity due to the presence of voids (Vs) formed during their development. There are a number of works conducted and reported on in this field [37,54,55,56,57,58,59,60,61,62,63,64,65,66,67,68,69].

## 4. Conclusions

This study demonstrates, for the first time, the feasibility of producing MOC-based heavyweight mortars using coarse hematite and barite aggregates of up to 8 mm for gamma radiation-shielding applications. The experimental findings reveal that the incorporation of coarse heavyweight aggregates significantly alters the internal packing structure of MOC mortars, which directly influences their mechanical, thermal, and radiation attenuation performance.

1. A linear relationship between the compressive and bending strengths was found when the compressive and bending strength values of mortar samples were analyzed.
2. The strength values of MOC samples produced with normal aggregate were higher than those with hematite and barite. Although the hematite and barite materials had a larger unit volume weight than the other samples, it is believed that this was because the materials’ structures caused microcavities in the samples that were produced, which prevented the materials from providing the required strength.
3. The thermal conductivity values of BMOC samples and HMOC samples gave similar results. The thermal conductivity values of mortars produced with normal aggregate were very high. Therefore, the thermal insulation values of samples with barite and hematite aggregates were significantly higher.
4. Although the heavyweight aggregates improved the radiation-shielding performance and reduced the thermal conductivity, the possible mismatch in the coefficients of thermal expansion between the aggregates and the MOC matrix may influence thermal damage resistance during repeated heating–cooling cycles. Future studies should therefore investigate the thermal shock resistance through elevated-temperature exposure and cyclic thermal loading tests.
5. The gamma attenuation results confirmed that the MOC binder itself contributes positively to radiation shielding compared to conventional Portland cement systems, and the use of barite aggregate increased the attenuation performance by more than 50%.
6. Although open pores were present, the pore structure consisted predominantly of closed pores. In addition, 5Mg(OH)_2_ and MgCl_2_ were detected in all samples. The formation of 5Mg(OH)_2_·MgCl_2_·8H_2_O (Phase 5) crystals was clearly observed. SEM images of the HMOC and NMOC samples confirmed the formation of Phase 5 magnesium oxychloride crystals. Additionally, hematite particles were also observed in the HMOC samples, which were evident by the glow on the SEM images. These observations indicate that hematite aggregate particles repel each other, creating minimal voids. Hematite aggregates exhibit elevated levels of porosity, a consequence of the presence of voids during the process of hematite formation.

MOC mortars containing hematite aggregates are shown to have strong thermal conductivity characteristics, while many other characteristics remain mostly the same. Hematite aggregates exert mutual pressure, resulting in the formation of voids inside the mortar. As a result of the gaps formed, its mechanical strength in thermal insulation is reduced. This work demonstrates that MOC cements, which normally utilize particles smaller than 2 mm, may effectively incorporate coarse heavyweight aggregates (8 mm) for radiation shielding without compromising their mechanical and thermal performance. Thus, it opens a new pathway for the development of alternative, environmentally favorable radiation-shielding construction materials as an alternative to Portland cement-based heavyweight mortars.

## Figures and Tables

**Table 1:** Mortar samples mix design (1 kg/m^3^).

Sample Code	MgO	MgCI_2_	A	H	B	Phosphate-Based Additive	Plasticizer	Additional Water
NMOC	425	313	1580	-	-	10.87	7.4	6.38
HMOC	425	313	-	1950	-	10.87	7.4	4.25
BMOC	425	313	-	-	2500	10.87	7.4	6.38

**Table 2:** Results of the pycnometer tests of the hematite, barite and normal aggregate.

Aggregate	ρ_a_	ρ_rd_	ρ_ssd_	WA_24h_
**Hematite**	3.484	3.317	3.365	1.445
	3.491	3.324	3.372	1.445
	3.495	3.320	3.370	1.503
**Average**	3.490	3.320	3.369	1.465
**Barite**	4.275	4.259	4.263	0.089
	4.292	4.277	4.281	0.082
	4.270	4.261	4.263	0.053
**Average**	4.279	4.266	4.269	0.075
**Normal aggregate**	2.704	2.687	2.693	0.227
	2.705	2.681	2.690	0.330
	2.708	2.687	2.695	0.282
**Average**	2.705	2.685	2.693	0.279
**WA_24h_**	Water absorption rate by mass in 24 h			
**ρ_a_**	Apparent grain density, g/cm^3^			
**ρ_rd_**	Oven-dry grain density, g/cm^3^			
**ρ_ssd_**	Saturated-surface dry grain density, g/cm^3^			

## Data Availability

The original contributions presented in this study are included in the article. Further inquiries can be directed to the corresponding author.
